# Novel Screening System of Virulent Strains for the Establishment of a *Mycobacterium avium* Complex Lung Disease Mouse Model Using Whole-Genome Sequencing

**DOI:** 10.1128/spectrum.00451-22

**Published:** 2022-05-17

**Authors:** Koji Furuuchi, Shintaro Seto, Hajime Nakamura, Haruka Hikichi, Akiko Miyabayashi, Keiko Wakabayashi, Kazue Mizuno, Teruaki Oka, Kozo Morimoto, Minako Hijikata, Naoto Keicho

**Affiliations:** a Department of Pathophysiology and Host Defense, The Research Institute of Tuberculosis, Japan Anti-Tuberculosis Association, Tokyo, Japan; b Department of Basic Mycobacteriosis, Nagasaki University Graduate School of Biomedical Sciences, Nagasaki, Japan; c Respiratory Disease Center, Fukujuji Hospital, Japan Anti-Tuberculosis Association, Tokyo, Japan; d Department of Clinical Laboratory, Fukujuji Hospital, Japan Anti-Tuberculosis Association, Tokyo, Japan; e Department of Pathology, Fukujuji Hospital, Japan Anti-Tuberculosis Association, Tokyo, Japan; f Vice Director, The Research Institute of Tuberculosis, Japan Anti-Tuberculosis Association, Tokyo, Japan; Johns Hopkins University School of Medicine

**Keywords:** *Mycobacterium avium* complex, mouse model, granuloma formation, whole-genome sequencing, SNVs, MAC-LD, granuloma

## Abstract

The establishment of animal models reflecting human Mycobacterium avium complex (MAC) lung disease (LD) pathology has the potential to expand our understanding of the disease pathophysiology. However, inducing sustained infection in immunocompetent mice is difficult since MAC generally shows less virulence and higher genetic variability than M. tuberculosis. To overcome this hurdle, we developed a screening system for identifying virulent MAC strains using whole-genome sequencing (WGS). We obtained nine clinical strains from Mycobacterium avium complex lung disease (MAC-LD) patients and divided them into two groups to make the mixed strain inocula for infection. Intranasal infection with the strain mixture of both groups in BALB/c mice resulted in progressive infection and extensive granuloma formation in the lungs, suggesting the existence of highly pathogenic strains in each group. We hypothesized that the change in the abundance of strain-specific single-nucleotide variants (SNVs) reflects the change in bacterial number of each strain in infected lungs. Based on this hypothesis, we quantified individual strain-specific SNVs in bacterial DNA from infected lungs. Specific SNVs for four strains were detected, suggesting the pathogenicity of these four strains. Consistent with these results, individual infection with these four strains induced a high lung bacterial burden, forming extensive peribronchial granuloma, while the other strains showed a decreased lung bacterial burden. The current method combining mixed infection and WGS accurately identified virulent strains that induced sustained infection in mice. This method will contribute to the establishment of mouse models that reflect human MAC-LD and lead to antimycobacterial drug testing.

**IMPORTANCE** To promote research on Mycobacterium avium complex (MAC) pathogenicity, animal models reflecting human progressive MAC lung disease (MAC-LD) are needed. Because there is high genetic and virulence diversity among clinical MAC strains, choosing a suitable strain is an important process for developing a mouse model. In this study, we developed a screening system for virulent strains in mice by combining mixed infection and whole-genome sequencing analysis. This approach is designed on the hypothesis that *in vivo* virulence of MAC strains can be examined simultaneously by comparing changes in the abundance of strain-specific single-nucleotide variants in the mouse lungs after infection with mixed strains. The identified strains were shown to induce high bacterial burdens and cause extensive peribronchial granuloma resembling the pulmonary pathology of human MAC-LD. The current method will help researchers develop mouse models that reflect human MAC-LD and will lead to further investigation of MAC pathogenicity.

## INTRODUCTION

The prevalence of nontuberculous mycobacterial lung disease (NTM-LD) is increasing worldwide and becoming a public health concern (1–3). Mycobacterium avium complex (MAC), which predominantly consists of M. avium and Mycobacterium intracellulare, is the most common etiology of NTM-LD in many countries, including Japan (4, 5). MAC lung disease (MAC-LD) comprises two representative radiological types, nodular bronchiectatic and fibrocavitary, with quite different clinical features (6). Recently, the incidence of nodular bronchiectatic MAC-LD, which is characterized by centrilobular nodules and bronchodilation in the middle lobe or lingula, has increased, especially among elderly women without preexisting lung disease or immunocompromised status (7, 8). Pathologically, cavity and bronchiectasis, the hallmarks of MAC-LD, are characterized by granulomatous inflammation throughout the airways (9, 10). Thus, granuloma formation is implicated in the pathogenesis of human MAC-LD.

Experimental mouse models have been widely used to study mycobacterial infections, including Mycobacterium tuberculosis and nontuberculous mycobacteria (NTM). Among the previous studies that have evaluated mouse models of NTM infection, mainly immunocompromised mice, such as beige and nude mice, were used (11, 12). However, the lack of reliable models that reproduce human-like lung disease has hindered a better understanding of the pathophysiology of NTM-LD (13). Since NTM are generally less virulent than M. tuberculosis, the difficulty in inducing a productive and sustained infection in immunocompetent mice is the current hurdle for the development of an experimental model (14, 15). In addition, because there is high genetic diversity (16, 17) and corresponding large diversity in virulence among MAC strains (18), choosing suitable strains could be an important process.

The virulence of mycobacteria, including M. tuberculosis and NTM, is known to be conditioned by a range of genes (19, 20). Recent advances in whole-genome sequencing (WGS) allow the high-resolution analysis of genetic variants ranging from single-nucleotide variants (SNVs) to large-scale deletions. SNVs could thus affect the bacterial phenotype (21), but these variants also could be used as a sort of barcodes to identify individual strains and may be useful to extract strains suitable for mouse models.

In this study, we developed a screening system for identifying pathogenic strains among multiple clinical MAC strains using mixed infection and WGS. The method allows us to examine the virulence of multiple strains simultaneously, contributing to the reduction in the number of animals needed for experiments, consistent with animal welfare (22). The identified strains were proven to have the ability to cause extensive peribronchial granuloma, which resembled the pathological finding in human MAC-LD (23). This method contributes to the establishment of mouse models that reflect human MAC-LD and leads to further investigation of MAC pathogenicity as well as antimycobacterial drug testing.

## RESULTS

### Obtaining MAC clinical strains and phylogenetic analysis.

**FIG 1:**
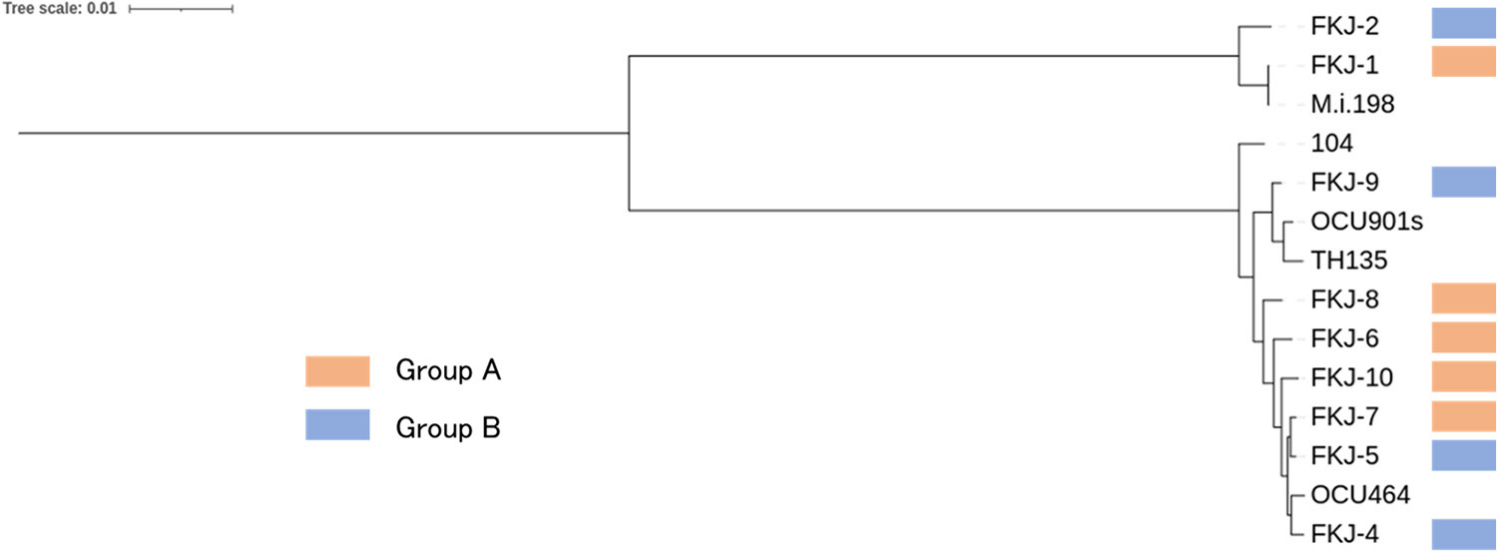
Phylogenetical tree of clinical MAC strains. Phylogenetic tree of the nine clinical strains in this study and five completely sequenced strains isolated from Japanese patients (OCU464, OCU901s, TH135, Mah104, and M.i. 198), constructed using variants after mapping with OCU464. Phylogenetic trees were constructed with the maximum-likelihood method using REALPHY software and then visualized in iTOL v6.

To select candidate pathogenic strains in mice, nine clinical strains, seven M. avium subsp. *hominissuis* and two M. intracellulare strains, from patients with progressive MAC-LD requiring treatment initiation were obtained. To examine the genetic relatedness between the nine strains and the representative strains of M. avium subsp. *hominissuis* and M. intracellulare in Japan, we carried out phylogenetic analysis including these nine strains and completely sequenced four MAC strains (OCU464, OCU901s, TH135, and M.i. 198) isolated from human immunodeficiency virus (HIV)-negative patients with MAC-LD and one M. avium subsp. *hominissuis* strain isolated from HIV-positive patients with disseminated infection (Mah104). The results showed that the nine strains were genetically related to the four strains isolated from MAC-LD patients and FKJ-1 was genetically very close to M.i. 198. Mah104 is most distantly related to all other M. avium subsp. *hominissuis* strains (Fig. 1).

### Change in bacterial loads after mixed MAC infection.

**FIG 2:**
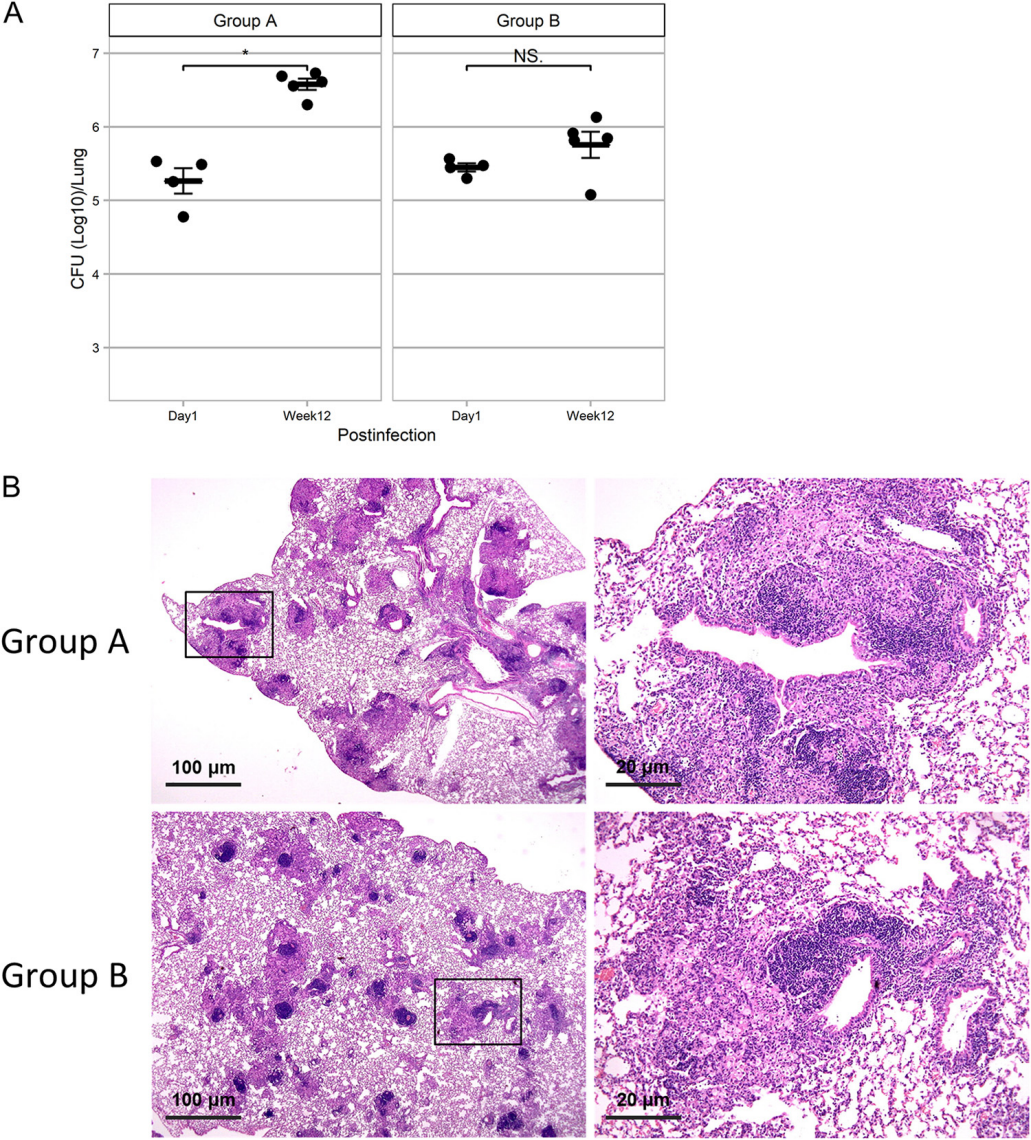
Bacterial burden and pathology of mouse lungs infected with mixed MAC strains. (A) Changes in CFU counts in the lungs after infection with mixed MAC strains of both groups. Data are presented as means and SEM. Asterisks represent statistical significance using the Mann–Whitney *U* test (*, *P* < 0.05; NS, not significant). (B) Representative pathological findings of the lungs at 12 weeks after infection with mixed MAC strains of both groups. Hematoxylin-and-eosin staining was used. Right images are enlarged images indicated by squares in the left panels.

To make the mixed strain inocula for infection, the nine clinical strains were divided into two groups (groups A and B). As shown in Fig. 1, group A included one M. intracellulare strain and four M. avium subsp. *hominissuis* strains, and group B included one M. intracellulare strain and three M. avium subsp. *hominissuis* strains. In each group, female BALB/c mice were infected with the strain mixture at a total dose of 1 × 10^6^ bacilli via the intranasal route. The bacterial number of mixed strains in infected lungs at 12 weeks postinfection (p.i.) was significantly increased in group A (*P < *0.05) and maintained in group B (*P = *0.19) compared with those at 1 day p.i. (Fig. 2A). Extensive granulomas composed of many epithelioid macrophages, including foamy macrophages and surrounding lymphocytes, were observed throughout the airways in both groups of mice at 12 weeks p.i. (Fig. 2B). These results suggested that both groups included highly pathogenic MAC strains for immunocompetent mice.

### Identifying candidate virulent strains by detecting and quantifying strain-specific SNVs.

**FIG 3:**
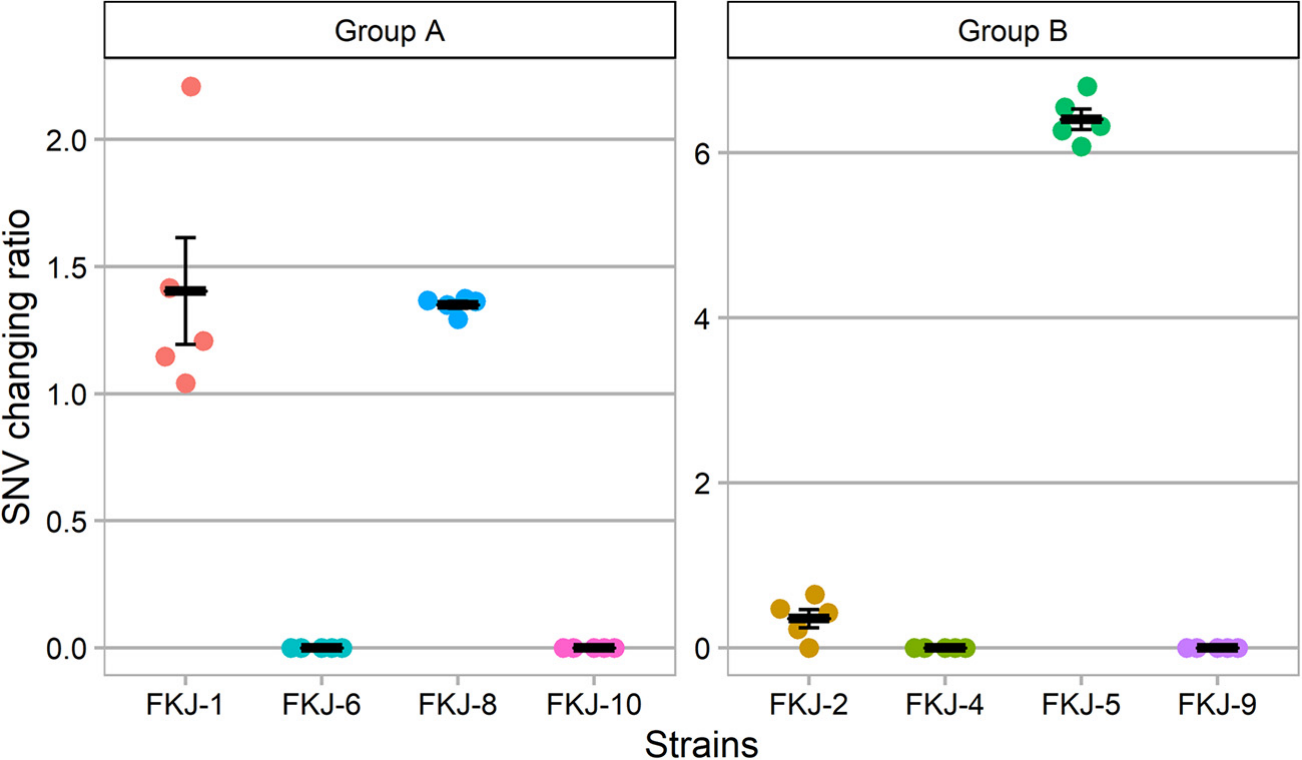
Changes in the proportion of strain-specific SNVs before and after infection. The SNV change ratio was calculated by dividing the proportion of each strain-specific SNV after infection by the proportion before infection. Data are presented as the means and SEM.

We hypothesized that the change in the bacterial burden of each strain in infected lungs can be estimated by the change in the abundance of strain-specific SNVs, which led to the screening of virulent strains due to the strong relationships between the *in vivo* growth and virulence observed in a previous study (18). Based on this hypothesis, bacterial DNA was extracted from cultures of inoculated mixed MAC suspensions (before infection) and lung tissue homogenates at 12 weeks p.i. (after infection), followed by WGS. The percentages of covered bases and mean coverages aligned to the OCU464 and mouse genomes of mixed culture samples are shown in Table 1, indicating that DNA extracted from cultures after infection was derived from MAC strains. The abundance of strain-specific SNVs in both samples was quantified by QuantTB (24). The specific SNVs for FKJ-1 and FKJ-8 in group A and for FKJ-2 and FKJ-5 in group B were detected in the infected lungs, whereas those for other strains were undetectable (Fig. 3). We then calculated the ratio of the change in the proportion of strain-specific SNVs before and after infection in each mouse. The mean change ratios of the number of strain-specific SNVs were 1.404 for FKJ-1 and 1.350 for FKJ-8 in group A and 0.354 for FKJ-2 and 6.405 for FKJ-5 in group B (Fig. 3). These results suggest that the four strains FKJ-1, FKJ-2, FKJ-5, and FKJ-8 were candidate virulent strains for BALB/c mice.

**TABLE 1:** The percentages of covered bases and mean coverages aligned to the OCU464 and mouse genomes of mixed culture samples

Group	Sample	OCU464		Mouse (mm10)	
		Percent covered	Mean coverage	Percent covered	Mean coverage
A	Before infection	98.5	107	0.000678	0.0000322
A	After infection, mouse 1	96.5	72.6	0.140	0.00686
A	After infection, mouse 2	96.5	67.9	0.109	0.00569
A	After infection, mouse 3	96.5	124	0.153	0.00365
A	After infection, mouse 4	96.5	115	0.0931	0.00285
A	After infection, mouse 5	96.5	104	0.0729	0.00219
B	Before infection	98.6	121	0.000789	0.0000385
B	After infection, mouse 1	97.0	128	0.0711	0.00214
B	After infection, mouse 2	98.0	115	0.0575	0.00219
B	After infection, mouse 3	97.8	120	0.0751	0.00225
B	After infection, mouse 4	97.0	123	0.0915	0.00265
B	After infection, mouse 5	98.0	113	0.0641	0.00194

### Bacterial quantification using real-time PCR.

**FIG 4:**
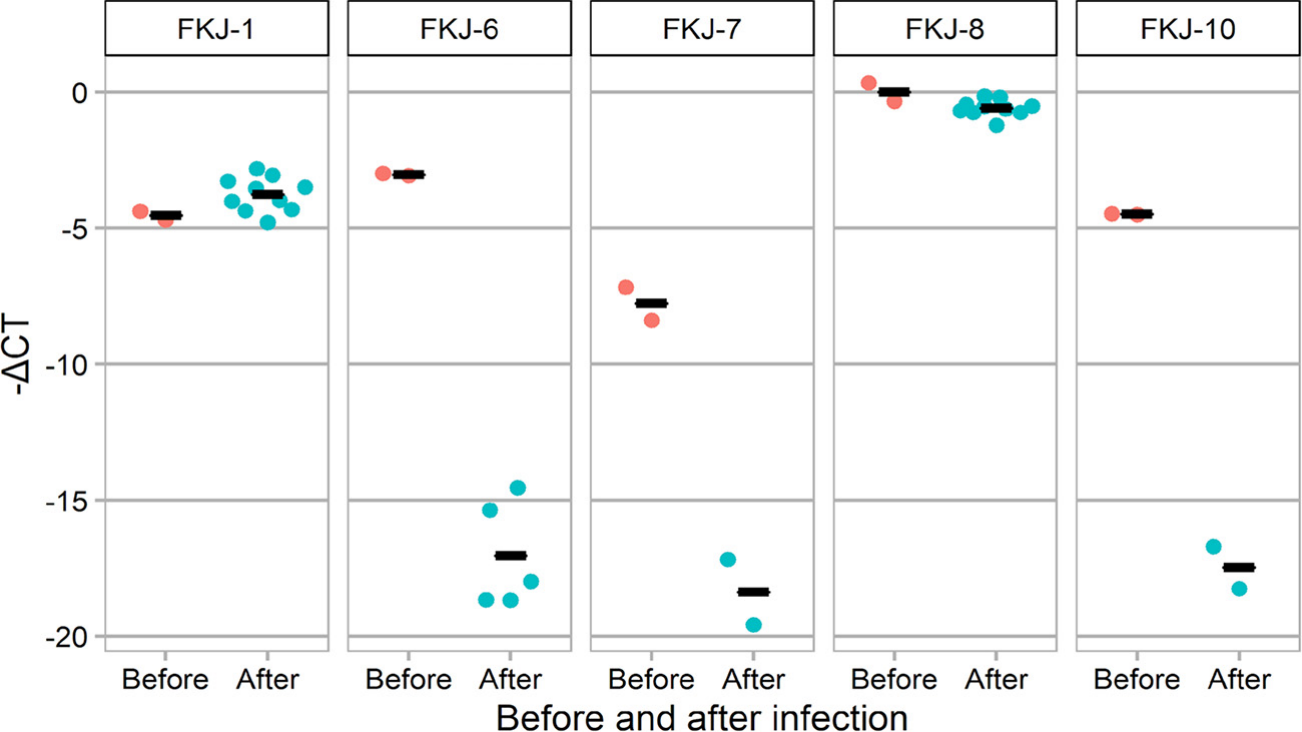
The relative abundance of each strain is represented as Δ cycle threshold (*Ct*) values before and after infection. The Δ*Ct* for each sample was determined by subtracting the *Ct* value of the target gene from that of the mycobacterial 16S rRNA gene of the same sample, that is, Δ*Ct* = *Ct*_(target DNA)_ – *Ct*_(mycobacterial 16S rRNA)_. Data are presented as the means and SEM.

Since FKJ-7 was not detected in the sample before infection (Fig. 3), we carried out real-time PCR for bacterial DNA from samples before and after infection to detect strains in group A (Fig. 4). Two strain-specific primer-probe sets for each strain in group A were constructed as shown in Table S1 in the supplemental material. In agreement with the results of whole-genome SNV analysis, the abundances of FKJ-6, FKJ-7, and FKJ-10 were significantly decreased after infection (*P < *0.05), with many decreasing to even undetectable levels, while those of FKJ-1 and FKJ-8 were maintained. Additionally, the abundance of DNA in FKJ-7 was lower than those in other strains, which would lead to undetectable SNV analysis in this strain.

### Lung pathology of mice infected with individual MAC strains.

**FIG 5:**
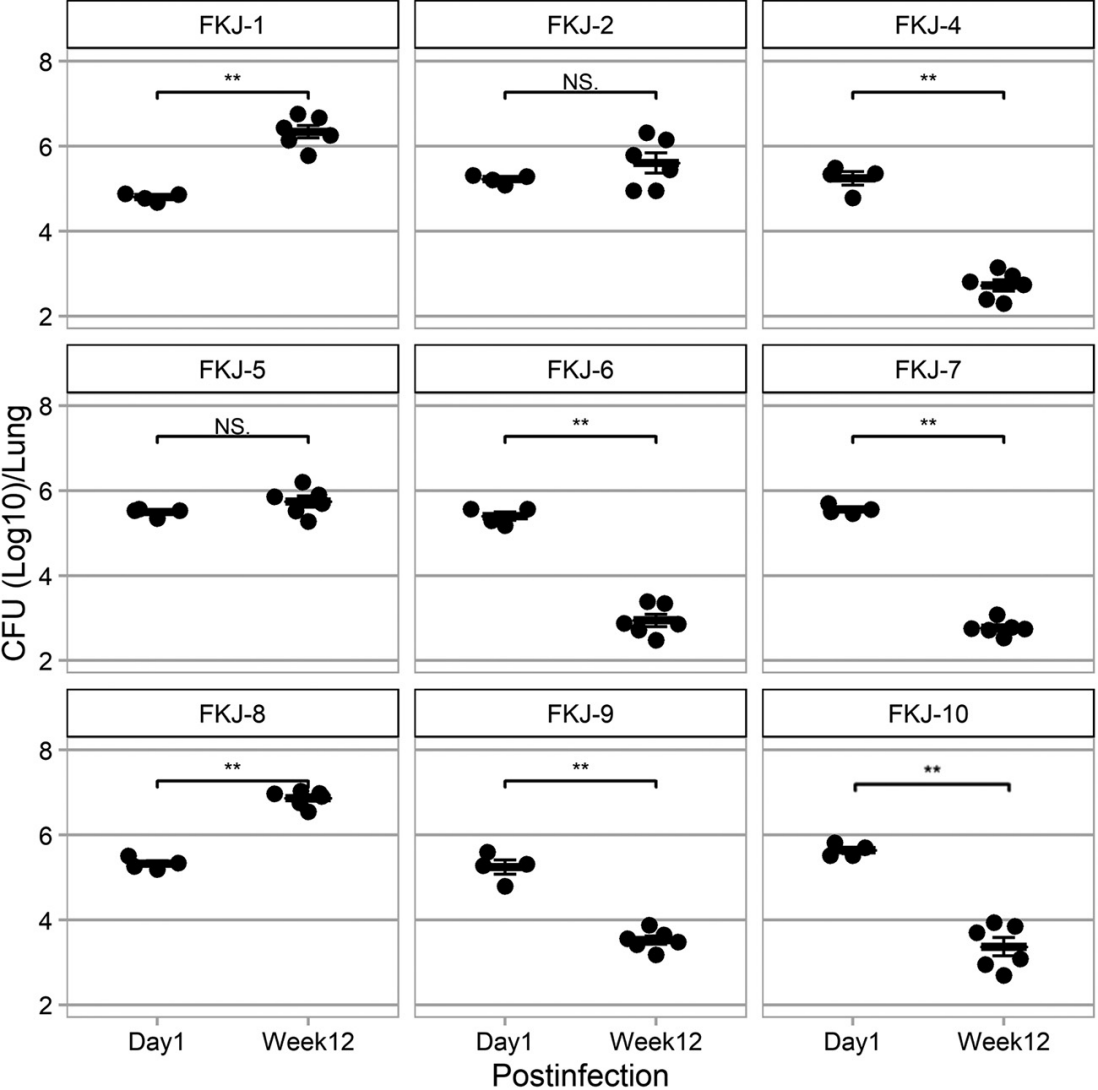
Changes in CFU counts in the lungs after infection with nine MAC strains individually. Data are presented as the means and SEM. Asterisks represent statistical significance using the Mann–Whitney *U* test (**, *P* < 0.01; NS, not significant).

**FIG 6:**
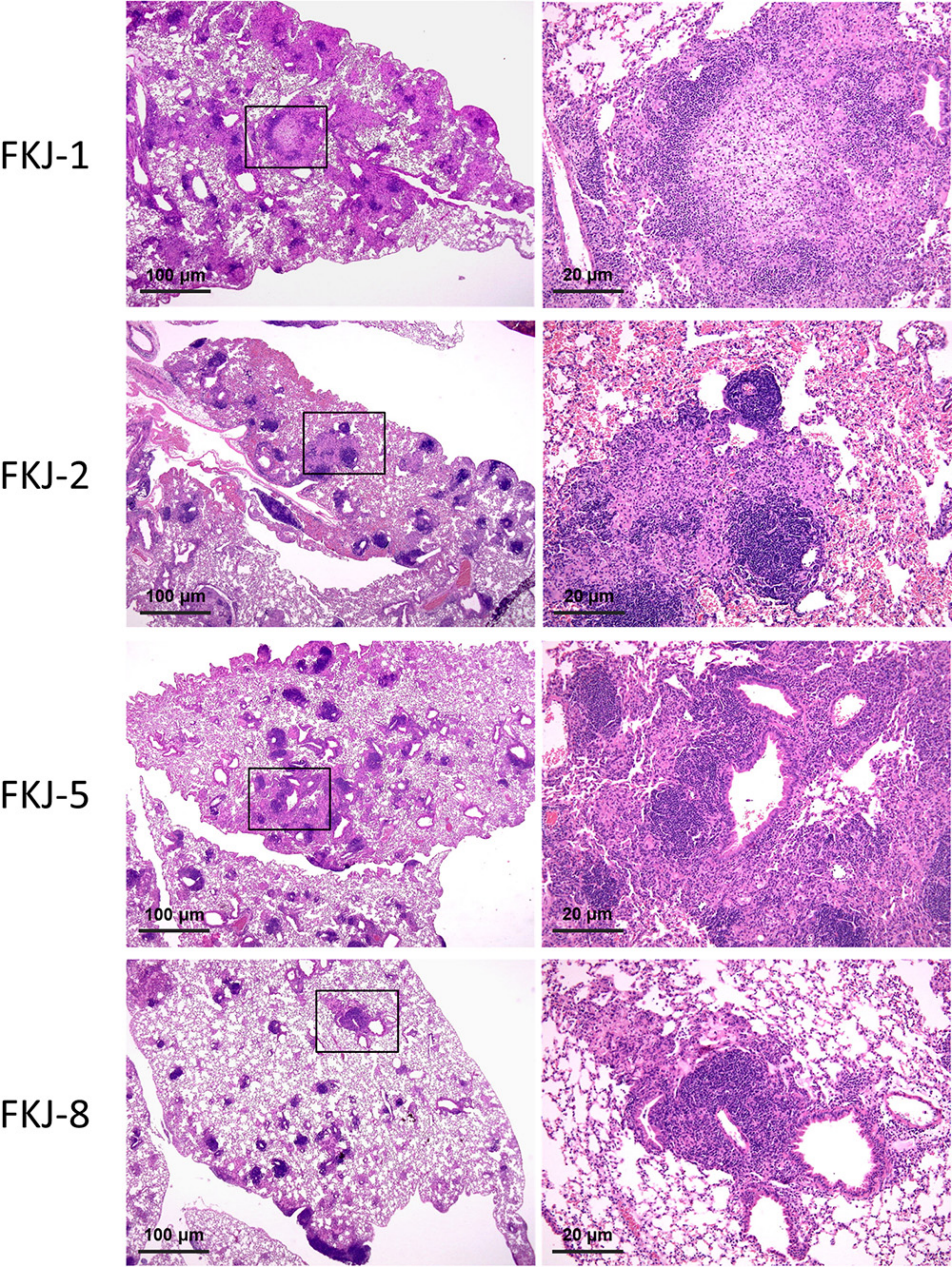
Representative pathological findings of the lungs at 12 weeks after infection with FKJ-1, FKJ-2, FKJ-5, and FKJ-8. Hematoxylin-and-eosin staining was used. Right images are enlarged images indicated by squares in the left panels.

To verify the above results, we infected nine clinical strains individually with mice. The lung bacterial loads at 1 day and 12 weeks p.i. are shown in Fig. 5. In agreement with the results of whole-genome SNV analysis, lung CFU at 12 weeks p.i. significantly increased in FKJ-1 (*P < *0.01) and FKJ-8 (*P* < 0.01) and were maintained in FKJ-2 (*P = *0.44) and FKJ-5 (*P = *0.33), while those of the other strain decreased significantly (*P < *0.01). Similarly, mice infected with the four strains exhibited extensive peribronchial unstructured granuloma, which resembles the pulmonary pathology in human MAC-LD (23) (Fig. 6), whereas there was no granuloma formation with only slight lymphocytic aggregation in the mouse lungs infected with other strains (Fig. S1). These results showed that the current method combining the mixed infection model and whole-genome SNV analysis accurately identified virulent strains with the ability to induce progressive infection in mice.

## DISCUSSION

Our understanding of the mechanism underlying MAC pathogenicity has been hindered by the lack of a universal animal model that reflects human progressive MAC-LD. Since MAC is generally less virulent than M. tuberculosis and has high genetic and virulence diversity (16–18), selecting a suitable strain is an important process for model establishment. In the present study, we developed a screening method for identifying virulent strains in immunocompetent mice by combining mixed infection and WGS analysis. This approach would overcome the difficulties in selecting clinical strains with variable virulence and could be an alternative to the method of testing virulence by passaging laboratory strains in mice. The primary strength of this method is that the *in vivo* virulence of multiple strains can be screened simultaneously by mixed infection, leading to a reduction in the number of animals required for experiments, consistent with animal welfare. The second strength is the high discrimination accuracy of whole-genome SNV analysis among multiple species or strains even with similar genetic features. The results clearly demonstrated that 4 of the 9 tested strains, including 2 M. avium subsp. *hominissuis* and 2 M. intracellulare strains, detected by SNV analysis showed high bacterial burdens in mouse lungs, while those of the other strains decreased. In addition, the four strains caused extensive peribronchial granuloma, which resembles the pulmonary pathology of human MAC-LD. The current method contributes to the establishment of mouse models of MAC infection that reflect human MAC-LD by facilitating the selection of suitable strains.

There is no standard method for establishing a mouse model of MAC-LD, as previous studies have used different strains, mice, infection doses, and routes of infection (14). In the present study, intranasal infection of immunocompetent mice with pathogenic MAC strains successfully caused progressive infection and extensive peribronchial granuloma, which characterizes the pulmonary pathology of human MAC-LD (10, 23). Fujita et al. evaluated the pathological findings of bronchiectasis in nine cases of MAC-LD using surgical specimens and proved extensive granuloma formation throughout the airway. In addition, destruction of bronchial cartilage and the smooth muscle layer and narrowing of the airway were caused by extensive submucosal granulomas (23). Since cartilage and smooth muscle play a major role in maintaining the airway lumen, destruction of these fundamental bronchial structures is likely the main mechanism for the development of bronchiectasis in human MAC-LD. Therefore, the result that the study mice presented similar pathological findings supports our methodology for establishing the mouse model reflecting human MAC-LD.

This study provides valuable insight into the virulence diversity of MAC clinical strains and the differences in virulence between MAH and MI. Phylogenetic tree analysis showed that the nine strains belong to the predominant strains in Japanese patients with MAC-LD. Among the clinical strains investigated, two of the seven MAH strains and both MI strains showed high virulence in BALB/c mice. Several studies have investigated the virulence of clinical MAC strains in mice. Tomioka et al. showed that non-HIV human-derived MI was more virulent than M. avium from non-HIV patients in C57BL/6 mice (25). Tateishi et al. investigated the virulence of six clinical MAC strains, including four M. avium and two M. intracellulare strains, using an intratracheal infection model and found that the M. intracellulare strains showed increased or maintained bacterial load, while the M. avium strains showed decreased bacterial load (18). Together with our results, these results indicate that MI from human MAC-LD could be more virulent in mice than M. avium subsp. *hominissuis*. In human MAC-LD, some studies suggested that M. intracellulare infection was associated with cavity formation and worse prognosis (26, 27), but the relative virulence of M. intracellulare in human remains unclear. Revealing the mechanism underlying the development of lesions in mice may also promote the research on their virulence in human. Among the two M. intracellulare strains, FKJ-1 exhibited higher bacterial loads and caused more progressive lung disease than FKJ-2. The phylogenetic tree showed that FKJ-1 is genetically very close to M.i. 198, which was proven to possess strong virulence in mice (18). Consistent with the high genetic diversity reported by Yano et al. (16), the seven M. avium subsp. *hominissuis* strains showed high virulence diversity, which indicates the involvement of strain virulence in the pathogenesis of human lung infection. However, considering that all MAH strains were isolated from patients with progressive disease, the phenotypic differences in disease severity between mice and humans could be partly explained by human host susceptibility. Indeed, almost all patients from whom MAH strains in this study were isolated were middle-aged to elderly, thin women, which represents the typical population susceptible to MAC-LD (28). To clarify the pathophysiology of MAC-LD, host-microbe interactions and host susceptibility factors need to be elucidated (29).

In our methods, BALB/c mice were infected with mixed multiple strains, and the individual MAC strains were identified using WGS analysis. Polyclonal or mixed infection was also commonly observed in human MAC-LD. Although the precise role is unclear, polyclonal infection was related to the nodular bronchiectatic form and environmental exposure (30, 31), and another study showed an association between polyclonal infection and disease progression (32). Furthermore, patients with MAC-LD often experience recurrence by either relapse from the same MAC strain or reinfection with a new strain (33). Given the ubiquitous MAC exposure, a process similar to this model, in which multiple strains comprising genetic and virulence diversity are competing and selected within the host (34), may be involved in the development of human NTM infection. The mixed infection model may be useful for elucidating the pathogenesis and mechanisms of polyclonal infection in humans. Additionally, in clinical settings, this WGS method could serve to distinguish relapse and reinfection and identify the source of infection.

For mixed infection, we divided nine strains into two groups to maintain the detection accuracy of WGS. The number of strains used for the mixed infection can be increased further, in principle, by analyzing more WGS reads. However, the accuracy may also depend on genetic similarities of the strains tested. Therefore, we should carefully adjust the bacterial number of strains in each experiment. Since the CFU of each strain at 1 day p.i. was not exactly the same in individual infections (Fig. 5), the proportion of strains at the mixed infection might not be equal. However, the fluctuation would not have a significant impact on our methodology, because virulent strains showed increment or detectable levels and avirulent strains were not detected in mouse lungs of mixed infection at 12 weeks p.i., which was consistent with the results of infection with individual strains (Fig. 3 and 5). In the WGS analysis, FKJ-7 was not detected in the sample before infection. The reduced sensitivity for SNV detection could be due to the difference in amount of DNA obtained through culture-dependent methods, as reported previously (35). Improving the detection power of WGS or applying the culture-independent method for DNA extraction would further improve the accuracy of the screening system. Since FKJ-5, which had a high SNV changing ratio in the mixed infection, showed almost unchanged lung CFU in individual infection, there is a possibility that the growth rates of the strain in the mixed and individual infections are not completely the same. However, because there was a substantial difference in the *in vivo* growth between virulent and avirulent strains (Fig. 5), the alteration of the strain behaviors would not have a significant effect on our objective of screening for virulent strains. The results that we detected only SNVs derived from the pathogenic strains from mouse lungs with mixed infection prove the validity of this approach as a screening method for the development of mouse models.

In conclusion, we developed a screening system for pathogenic strains among multiple clinical MAC strains by combining mixed infection and WGS, which accurately identified virulent strains in immunocompetent mice. This method will contribute to the establishment of a mouse model that reflects human MAC-LD and will lead to further investigation of MAC pathogenicity. Furthermore, the method could be applicable for other important pathogens, such as the Mycobacterium abscessus complex, a less virulent but emerging pathogen.

## MATERIALS AND METHODS

### Ethics statement.

The animal experiments in this study were approved by the Animal Care and Use Committee of The Research Institute of Tuberculosis (permit number: 2020-01). Animals were treated in accordance with the ethical guidelines of the Research Institute of Tuberculosis, Japan Anti-Tuberculosis Association.

### Mycobacterial strain and culture conditions.

First, we examined the virulence of a representative clinical strain in Japan in mice, OCU901s, an MAH strain isolated from a Japanese immunocompetent patient with MAC-LD (16), which was kindly gifted by Yukiko Nichiuchi, Toneyama Institute for Tuberculosis Research, Osaka City University Medical School. However, the strain showed low virulence and failed to establish chronic infection in BALB/c mice with intranasal infection at a dose of 1 × 10^6^ CFU at 12 weeks p.i. (Fig. S2 in the supplemental material). Then, 10 MAC isolates were obtained from sputum specimens of patients with progressive MAC-LD requiring treatment initiation who were visiting the Fukujuji Hospital, Japan Anti-Tuberculosis Association in Tokyo, Japan. All patients met the diagnostic criteria of the current 2020 guidelines for the treatment of NTM-PD (36). Of the 10 isolates, 1 isolate was excluded because it was confirmed to include two distinct strains by WGS analysis; therefore, the remaining nine clinical strains were used. The designation of the nine strains and the characteristics of patients from whom the strains were isolated are summarized in Table S2. The strain names are derived from the name of the hospital in which they were isolated. The radiographic features on computed tomography were classified into fibrocavitary, cavitary nodular bronchiectatic, and noncavitary nodular bronchiectatic (37). All strains were cultured in 7H9 medium (BD Biosciences, San Jose, CA) supplemented with 10% albumin-dextrose-catalase (ADC), 0.5% glycerol, and 0.05% Tween 80 at 37°C. Single-cell suspensions of each strain were prepared as described previously (38), and CFU of MAC clinical strains were determined on Middlebrook 7H10 agar plates supplemented with 10% oleic acid-albumin-dextrose-catalase (7H10).

### DNA extraction.

For genomic DNA preparation of each MAC strain, the cell suspension was incubated on 7H10 for 2 weeks at 37°C. Similarly, genomic DNA of mixed strains before and after infection was prepared by culturing inoculated mixed MAC suspensions (before infection) and whole lung cell suspensions at 12 weeks p.i. on 7H10 for 2 weeks at 37°C. For preparation of lung cell suspensions, whole lung lobes were homogenized with ShakeMaster Neo (Bio Medical Science). Genomic DNA was extracted as described previously, with slight modification (39). Briefly, bacteria obtained from 7H10 were suspended in Tris-EDTA (TE) buffer and then treated with an equal volume of chloroform/methanol (2:1, vol/vol). After rocking for 2 min, cell suspensions were centrifuged, followed by the removal of aqueous and organic phases to collect bacterial pellets. The delipidated bacteria were dried at 55°C for 60 min and then resuspended with TE containing lysozyme (Wako Pure Chemical Industries, Osaka, Japan) at 1 mg/mL. After treatment with lysozyme at 37°C for more than 12 h, 1/10 volume of 10% SDS (wt/vol) and proteinase K (FUJIFILM Wako Pure Chemical Industries, Osaka, Japan) at 0.2 mg/mL were added. Tubes were then mixed by gentle inversion and incubated at 50°C for 3 h in a dry bath. Cell suspensions were treated with an equal volume of phenol-chloroform/isoamyl alcohol (PCI; 25:24:1, vol/vol) and then subjected to isopropanol precipitation. Precipitated genomic DNA was treated with 100 μg/mL RNaseA for 30 min at 37°C, followed by PCI treatment and ethanol precipitation. Purified genomic DNA was suspended in TE buffer.

### Determination of genome sequences of each strain and phylogenetic analysis.

Libraries were prepared from genomic DNA from nine individual MAC strains and mixed bacteria in infected mouse lungs with a QIAseq FX DNA library kit (Qiagen). In the library preparation for each MAC strain and mixed bacteria from infected mouse lungs, PCR was performed for two cycles with 300 ng of genomic DNA and five cycles with 50 ng of genomic DNA. Libraries were sequenced using Illumina MiSeq (Illumina) and paired-end sequencing (250 bp for read 1 and 250 bp for read 2). Mapping was performed using bwa-mem v0.7.15 to the complete genome sequences of OCU464 (accession number CP009360) (16). Phylogenetic trees were constructed with the maximum-likelihood method using REALPHY software (version 1.13) (40). Briefly, the sequences were mapped to the reference sequence (OCU464 genome) via Bowtie2 (41). From these initial alignments, multiple sequence alignments were recreated using PhyML for tree construction (42). The tree was then visualized in iTOL v6 (43). We included complete genome sequences of representative clinical strains from Japanese patients as references, which are expected to be genetically close to tested strains in the phylogenetic tree. These strains were OCU464 (accession number NZ_CP009360), OCU901s (accession number NZ_CP018014), TH135 (accession number NZ_AP012555), and M.i. 198 (accession number NZ_AP024265). We also used Mah104 (accession number NC_008595) as another reference, isolated from an AIDS patient in the United States.

### MAC infection in mice.

Specific pathogen-free female BALB/c mice were purchased from Japan SLC Co. Ltd. (Shizuoka, Japan). Mice were maintained in the isolation cabinet (Tokiwa Kagaku Kikai, Tokyo) installed in the animal biosafety level II facilities (ABSL-2) at the Research Institute of Tuberculosis, Japan Anti-Tuberculosis Association. Female mice (6 weeks old) were infected with each MAC strain or the mixture containing four to five strains via the intranasal route at a dose of 1 × 10^6^ CFU in 30 μL of saline. For mixed infection, each strain was prepared at equal concentration in the mixed inocula.

### CFU assay.

The inoculum dose was confirmed by a CFU count of mouse lung tissue homogenates obtained 24 h p.i. The infected lungs were obtained at 12 weeks after infection for evaluation of the bacterial loads and histopathological analysis. Bacterial counts were determined by plating serial dilutions of whole lung homogenates of individual mice onto Middlebrook 7H10 agar plates. CFU were quantified after incubation for 3 weeks at 37°C.

### Histological analysis.

The whole lung lobes from each mouse were fixed with 10% formalin in phosphate-buffered saline (PBS). Sections from these tissues were stained with hematoxylin and eosin to visualize tissue alterations. Localization of the lesions was confirmed by Elastica van Gieson staining.

### Quantifying strain-specific SNVs using QuantTB.

To quantify individual MAC strains, we used QuantTB (24), a method to identify and quantify individual M. tuberculosis strains in WGS data, changing a reference to the complete genome sequence of OCU464 (accession number CP009360). Briefly, QuantTB predicts the combination of strains from the allelic variation observed in a sample using SNV markers.

### Bacterial quantification by real-time PCR for bacterial DNA.

Specific primer-probe sets for mycobacterial 16S rRNA and for each clinical strain were designed utilizing CLC Genomic Workbench (CLC Bio, Cambridge, MA), as shown in Table S1. Quantitative real-time PCR was performed using a StepOnePlus (Applied Biosystems) and the following optimized cycling conditions: one cycle of 95°C for 10 min followed by 45 cycles of 95°C for 30 s and 60°C for 1 min. TaqMan Universal master mix II, no UNG (Applied Biosystems) was used for PCRs. The resultant real-time PCR data were analyzed using StepOne software V2.3 (Applied Biosystems). Probes with cycle threshold (*Ct*) values of 45 or greater were considered undetectable. In statistical analysis, undetectable *Ct* values were replaced by imputed values generated by adding a random number less than 1 to the detected maximum *Ct* value. To calculate the relative abundance of each strain, the Δ*Ct* for each sample was determined by subtracting the *Ct* value of the target gene from that of the mycobacterial 16S rRNA gene of the same sample, that is, Δ*Ct* = *Ct*_(target DNA)_ – *Ct*_(mycobacterial 16S rRNA)_.

### Statistical analysis.

Data are presented using the mean values plus or minus the standard error of the mean (SEM). The Mann–Whitney *U* test was used to evaluate the differences in CFU and Δ*Ct* values between 1 day and 12 weeks after infection. A *P* value of less than 0.05 was considered significant. All statistical analyses were performed using R version 3.5.1.

### Data availability.

These raw sequence data have been deposited in the DRA database under the accession number DRA013277.
