# Supplementary material for: Novel Screening System of Virulent Strains for the Establishment of a Mycobacterium avium Complex Lung Disease Mouse Model Using Whole-Genome Sequencing
Source: Microbiol Spectr. 2022 May 17;10(3):e00451-22. doi: 10.1128/spectrum.00451-22 (PMC9241706; doi:10.1128/spectrum.00451-22)
Supplement: SUPPLEMENTAL FILE 1 — Supplemental material. Download spectrum.00451-22-s0001.pdf, PDF file, 0.7 MB [file spectrum.00451-22-s0001.pdf]

Supplementary Figure legends

Supplementary Figure S1. Representative pathological findings of the lungs at 12 weeks after infection with FKJ-4, FKJ-6, FKJ-7, FKJ-9, and FKJ-10. Hematoxylin-and-eosin staining was used. Right images are enlarged images indicated by squares in the left panels.

Supplementary Figure S2. Bacterial burden and pathology of mouse lungs infected with MAH OCU901s.

(A) Changes in CFU counts in the lungs after infection with OCU901s. Data are presented as the means and SEM. Asterisks represent statistical significance using the Mann–Whitney U test (**$p < 0.01$; NS, not significant).

(B) Representative pathological findings of the lungs at 12 weeks after infection with OCU901s. Hematoxylin-and-eosin staining was used. Right images are enlarged images indicated by squares in the left panels.

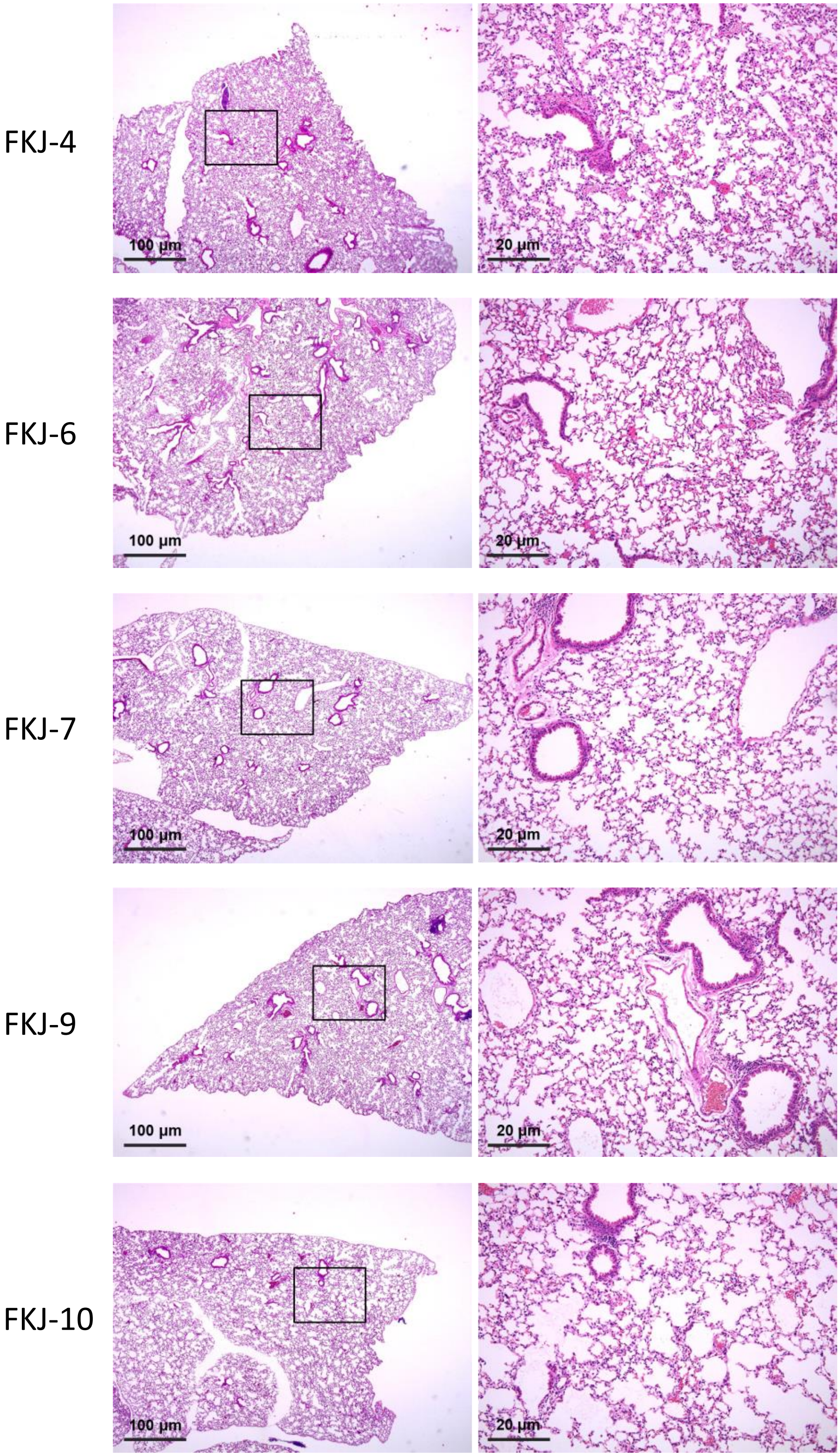
FKJ-4
100 μm
20 μm
FKJ-6
100 μm
20 μm
FKJ-7
100 μm
20 μm
FKJ-9
100 μm
20 μm
FKJ-10
100 μm
20 μm

Fig. S1

A

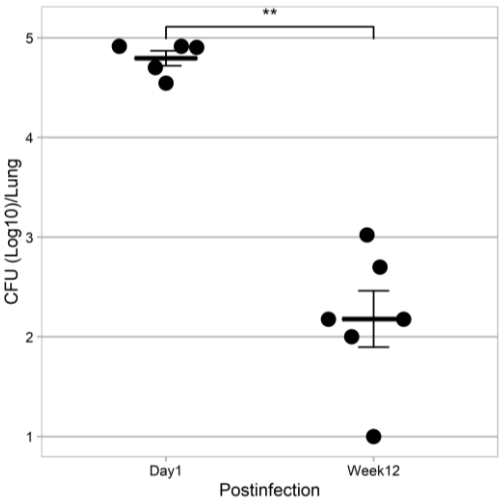

B

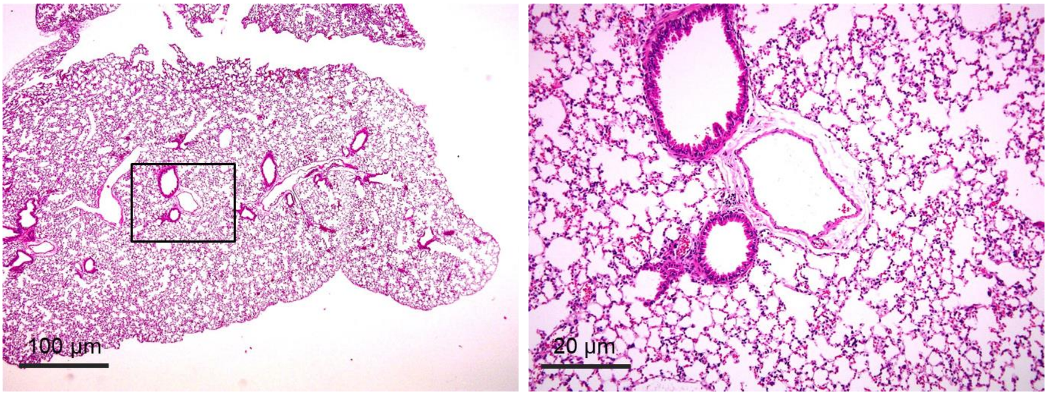

Fig. S2

Supplementary Table S1

Real-time PCR assays for detection of each MAC strain in the Group A

Target region	Forward primer (5' to 3')	Reverse primer (5' to 3')	Probe (5' to 3')
16s rRNA	CCGGAATTACTGGGCGTAAA	AGTACTCTAGTCTGCCCGTATC	TTCACGAACAACGCGACAAACCAC
FKJ-1_1	CCCGCTACGACGATGAATTA	GATGTTCGTGTCGAGGTAGAG	TCTTCACCAAGCGGCATTACTCGA
FKJ-1_2	CGCTGCAGGACTGGATTTAT	GATCTGGTGATTGAGGTGATCG	TCATGAGCCGCCTTCCATCAAGTC
FKJ-6_1	CTCGGATCGATGGCGTATTT	CGAATGATCTGGGTCACTCATC	TCTAACGAGTTGTTCCTCGCCGG
FKJ-6_2	TTCGCAGCATGACCGATAA	GTAGAGGTTCAGTTCGTGGTAG	TACGCACCTGATGCAGAAGCTTGA
FKJ-7_1	AGTATCGCGCACCAGTATTT	TTCGTGCTGTCATTACCTAGAC	ACTTGTGAACTACCTCTGCGGCAA
FKJ-7_2	GAGGCACACCTGGATGTATTT	AGCCAAAGAATATGCGGATCAA	AGCGTTGGGTCATCCGCATATTCT
FKJ-8_1	TAAGCGTGCCGAGGTTTAC	GAGGGAGTCGTCGTACAGATA	AGGCACAGTTCAAGAAGCGTCGT
FKJ-8_2	CTGGCTACACGGTCTTTCATAG	CTCTTTCGGTTCTCCCGAATC	TATCGCCATGCGTACACTTCCTGC
FKJ-10_1	CGACAGATGAATGCCCAGATAG	GCGGCGAGAAGTCCATAAT	CGGTTCGTCCTCGGTAAAGCCTTC
FKJ-10_2	CAAGGTAGTCTCGCTGTTCTC	GTTTCGCAGAACTGGCTTTC	CAGGCGGGTTGGATGTTGGTCTA

Supplementary Table S2

MAC strains and characteristics of patients from whom the strains were isolated.

Strains	Species	Age	Sex	BMI	Radiological features	Sputum smear
FKJ-1	*M. intracellulare*	70	Female	16.6	Cavitary NB	1+
FKJ-2	*M. intracellulare*	65	Female	22.5	Cavitary NB	2+
FKJ-4	MAH	64	Female	20.4	Cavitary NB	±
FKJ-5	MAH	58	Female	17.1	Cavitary NB	1+
FKJ-6	MAH	76	Female	17.6	Noncavitary NB	1+
FKJ-7	MAH	54	Male	18.0	FC	±
FKJ-8	MAH	72	Female	17.6	Noncavitary NB	±
FKJ-9	MAH	63	Female	20.6	Cavitary NB	±
FKJ-10	MAH	43	Female	20.1	Noncavitary NB	1+

BMI, body mass index; FC, fibrocavitary; NB, nodular bronchiectatic; MAC, *Mycobacterium avium* complex; MAH, *Mycobacterium avium* subsp. *hominissuis*
